# Immunoproteomic Analysis of *Dirofilaria* *repens* Microfilariae and Adult Parasite Stages

**DOI:** 10.3390/pathogens10020174

**Published:** 2021-02-05

**Authors:** Anna Zawistowska-Deniziak, Katarzyna Powązka, Mateusz Pękacz, Katarzyna Basałaj, Maciej Klockiewicz, Marcin Wiśniewski, Daniel Młocicki

**Affiliations:** 1 Witold Stefański Institute of Parasitology, Polish Academy of Sciences, 00-818 Warsaw, Poland; katarzyna.powazka0@gmail.com (K.P.); pekaczmateusz@gmail.com (M.P.); kbasalaj@twarda.pan.pl (K.B.); danmlo@twarda.pan.pl (D.M.); 2 Division of Parasitology, Department of Preclinical Sciences, Faculty of Veterinary Medicine, Warsaw University of Life Sciences-SGGW, 02-786 Warsaw, Poland; maciej_klockiewicz@sggw.pl (M.K.); marcin_wisniewski@sggw.edu.pl (M.W.); 3 Department of General Biology and Parasitology, Medical University of Warsaw, 02-004 Warsaw, Poland

**Keywords:** dirofilariosis, antigens, host-parasite interactions, gene cloning, microfilaria, nematode

## Abstract

*Dirofilariarepens* is a parasitic nematode causing a vector-borne zoonotic infection (dirofilariosis), considered an emerging problem in human and veterinary medicine. Currently, diagnosis is based on the detection of the adult parasite and microfilariae in the host tissues. However, the efficacy of tests relying on microfilariae detection is limited by microfilariae periodic occurrence. Therefore, a new reliable and affordable serological diagnostic method is needed. Better characteristic of the parasite biology and its interaction with host immune system should help to achieve this goal. This study analyzes adult and microfilariae proteomes, and the use of one-dimensional electrophoresis (1-DE) and two-dimensional electrophoresis (2-DE) proteomics, immunoproteomics, and LC-MS/MS mass spectrometry allowed us to identify 316 potentially immunogenic proteins (75 belong to adult stage, 183 to microfilariae, and 58 are common for both). Classified by their ontology, the proteins showed important similarities and differences between both parasite stages. The most frequently identified proteins are structural, metabolic, and heat shock proteins. Additionally, real-time PCR analysis of some immunogenic targets revealed significant differences between microfilariae and adult life stages. We indicated molecules involved in parasite-host interactions and discussed their importance in parasite biology, which may help to reveal potential diagnostic antigens or select drug and vaccine targets.

## 1. Introduction

*Dirofilaria repens* (*Onchocercidae*) is a parasitic nematode causing dirofilariosis, an increasingly diagnosed zoonosis [1,2,3] spread by mosquitos [4,5]. The definitive hosts of the parasite are mainly carnivorous, especially canines; however, human infections have appeared more often in recent years. Until recently, scientist thought that humans could be an accidental host. Ermakova et al. [6] have found that only 10.5% and 0.9% of examined patients were positive for mature female or male worms, respectively. However, several reports indicate completion of the parasite life cycle in humans and the presence of microfilaremia [7,8,9]. Therefore, dirofilariosis is now recognized as a significant vector-borne human health risk factor. The lack of rapid and reliable diagnostic tools, along with inadequate knowledge of parasite biology, make it challenging to limit the spread of this zoonosis, especially while a vaccine is unavailable. Currently, the only way to reduce the risk of infection is the development of the diagnostic methods, specific not only for humans but also for dogs, the main reservoir of the parasite.

Human *D. repens* infections occur prevalently in Southern Europe, Asia, and occasionally Africa, and the north border of parasitosis in Europe was Cherbourg in France [10]. Still, as a result of climate change and human activities (such as air and road travel, shipment of goods resulting in the spread of invasive parasites, animal transport, travelling with pets), *D. repens* infections spread through Central and Eastern part of Europe, especially Poland, Lithuania, Ukraine, and Slovakia [2,3,11,12,13,14,15,16,17,18,19].

The number of infected dogs and humans are continuously increasing all over Europe, and studies report around 3500 cases of human dirofilariosis between 1977 and 2016 throughout the continent [3]. Moreover, the growing risks of human dirofilariosis in travelers have been discussed [20]. Surprisingly, despite its emergence and high zoonotic potential, researchers give less attention to *D. repens* when compared to *Dirofilaria immitis*. Although these two parasites share evident genetic similarities and common vectors, *D. repens* shows higher zoonotic potential than closely-related *D. immits.* This results in a higher prevalence of human subcutaneous rather than pulmonary dirofilariosis, even in the areas where *D. immitis* dominates over *D. repens* in canine populations [21]. The reason for the different zoonotic potential between these two species is puzzling, and we can only hypothesize that *D. repens* migration to subcutaneous tissues provides better conditions for escaping the host’s immune response. Additionally, the rate of undiagnosed dogs continuing an uncontrolled spread of the parasite might also be to blame. The symptoms of subcutaneous dirofilariosis are blurry and scanty. The most characteristic indicator is the formation of the subcutaneous nodules in which encapsulated parasites hide from the host’s immune reaction.

Additionally, the adult *D. repens* worms can be present in other tissues, including the abdominal wall, vitreous body of the eye, eyelid, seminal cord, scrotum, or testicle [22,23,24]. More unusual parasite locations are connective tissue around the elbow appendix [25] and tongue [26]. However, it seems that the most common form of human dirofilariosis is ocular dirofilariosis [2,27], and a few reported migrating larvae syndrome [27,28].

Currently, diagnosis of the infection is based on the detection of an adult parasite in the affected tissue and microfilariae detection by a modified Knott’s method or multiplex PCR [29,30]. However, tests involving microfilaria recognition are limited by the periodicity of microfilariae occurrence in the host [31]. A proper diagnosis of infection is problematic as veterinarians and medical doctors are not always aware of possible infection due to unspecific symptoms of the disease. The most reasonable solution is the development of new reliable and affordable serological diagnostic tools. To achieve this goal, we need to better characterize the biology of the parasite and its interaction with the host immune system.

Our study aimed to identify immunoreactive proteins present in both adult worms and microfilariae of *D. repens* that could be of a potential diagnostic value or useful as a drug or vaccine targets. The study allowed us to identify molecules common for both microfilariae and the adult stage, but also the stage-specific molecules. To our knowledge, a comparative analysis of *D. repens* immunoproteomes has not been performed before and therefore the obtained data are unique in this sense. Additionally, we cloned cDNA of the gene coding proteins identified in immunoreactive spots and measured gene expression levels in both parasite stages.

## 2. Results

### 2.1. One-Dimensional Electrophoresis (1-DE) Analysis of D. repens Microfilariae and Adult Worm Immunoreactive Proteins

**Figure 1:**
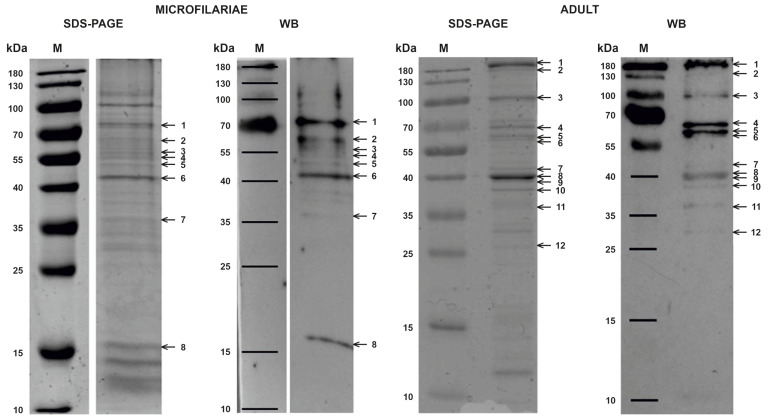
Comparative results of SDS–PAGE and Western Blot of extracts collected from microfilariae and adult *D. repens* (*Dirofilaria repens*) nematodes.

SDS–PAGE and Western Blot analysis of microfilariae and adult *D. repens* worm extracts revealed bands immunoreactive with serum of an infected dog: 8 for microfilariae and 12 for the adult stage (Figure 1, Supplementary Files S1 and S2). A similar band pattern was visualized in each of the three repeats.

Liquid chromatography and tandem mass spectrometry (LC-MS/MS) analysis of immunogenic regions allowed us to identify 287 proteins: 223 for microfilariae and 107 for the adult stage. Among microfilariae proteins, the most frequently identified were actin (8 bands), ATP synthase (5 bands), and heat shock protein 70 (5 bands), while in the adult parasite, the most frequently identified were actin (11 bands), calponin (5 bands), heat shock protein 70 (5 bands), intermediate filament protein (11 bands), and major antigen (5 bands).

The strongest signal on Western Blot of the microfilariae extract was observed in areas 1, 2, 6, and 8, and from these regions, 70 different proteins were annotated, whereas, for the adult worm, the clearest signal was associated with bands 1, 4, and 5, where almost 40 potential proteins were predicted.

### 2.2. Two-Dimensional Electrophoresis (2-DE) Analysis of D. repens Microfilariae and Adult Worm Immunoreactive Proteins

**Figure 2:**
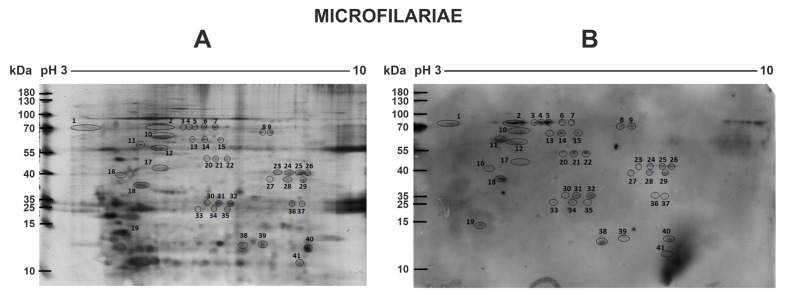
2-DE silver stained protein spot map of the extract from microfilariae *D. repens* (**A**); 2-D immunoblot showing potentially immunogenic protein spots (**B**). Immunogenic spots analyzed by mass spectrometry are circled and numbered.

**Figure 3:**
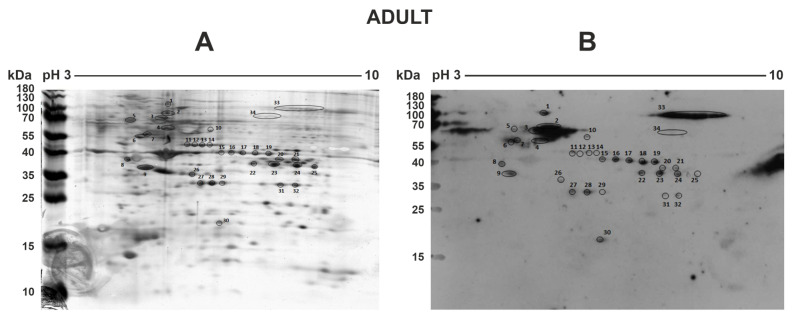
2-DE silver stained protein spot map of the extract from adult *D. repens* (**A**); 2-D immunoblot showing potentially immunogenic protein spots (**B**). Immunogenic spots analyzed by mass spectrometry are circled and numbered.

The two-dimensional electrophoresis (2-DE) study showed a pattern of microfilariae and adult stage proteomes with spots located between 10–130 kDa and in a pH range of 3–10 (Figure 2 and Figure 3, Supplementary Files S3 and S4). 2-D immunoblot analysis revealed 34 and 41 spots positively recognized by sera from dogs naturally infected with *D. repens* in microfilariae and the adult stage, respectively.

As shown in Figure 2, most of the potentially immunogenic proteins of the microfilariae stage migrated with molecular weight (MW) of 10 to almost 100 kDa and were located in areas with a pH range of 5–9.

In the adult stage (Figure 3, Supplementary File S4), most immunogenic proteins were distributed in MW between 15 and 130 kDa and located in the similar regions of pH 5–9.

### 2.3. LC-MS/MS Identification of Immunogenic Proteins of D. repens

LC-MS/MS analysis of spots excised from microfilariae and the adult stage, respectively, detected 316 potentially immunogenic proteins: 183 for microfilariae, 75 for the adult stage, and 58 common for both stages. Several proteins were identified in multiple spots with a different MW and pH, which may suggest that they underwent post-translational modifications. In most of the spots, more than one protein was identified. The protein most frequently found in different spots of the adult stage was actin (in 22 spots), whereas for microfilariae, it was heat shock protein 70 (7 spots) and intermediate filament tail domain protein (6 spots). Analysis of proteins detected by two methods, 1-DE and 2-DE, revealed molecules that were stage-characteristic and common for both (Table 1). Proteins specific for adult parasites and detected with two techniques were, e.g., LFI-1 protein, major antigen, spectrin protein 1, and, for microfilariae, e.g., ATP synthase subunit alpha, Bm13662 isoform a, chaperon protein DnaK, and KH domain protein. In several spots from 2-D gels from microfilariae, we identified individual proteins (spots number: 1, 18, 23–26, 40).

### 2.4. Gene Ontology of the Potentially Immunogenic Proteins of D. repens

**Figure 4:**
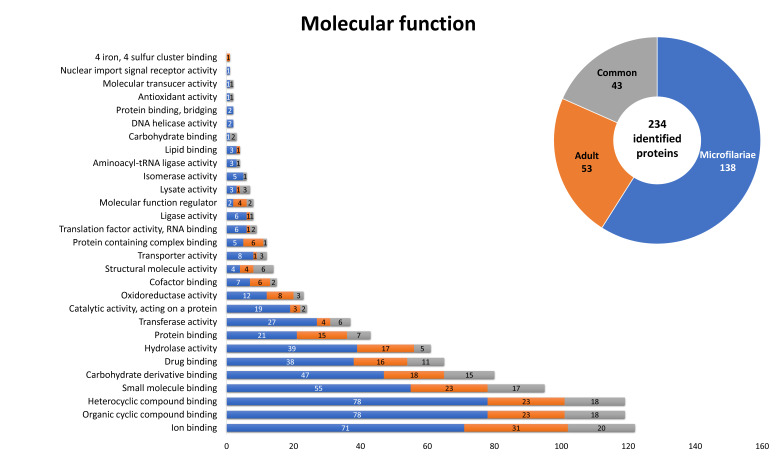
Proteins identified in both stages of *D. repens*, classified by their molecular functions, according to gene ontology (GO), The information was obtained from UniProtKB databases.

According to bioinformatic evaluation, 234 of 316 identified proteins were assigned by their molecular function (Figure 4). A total of 138 were characteristic for microfilariae, 53 for the adult stage, and 43 were common for both stages. A large number of proteins assigned for both stages was associated with organic and heterocyclic compound binding, ion binding, small molecule binding, carbohydrate derivative binding, and hydrolase activity. Most of these proteins were related to cellular (29 proteins) and metabolic processes (17). Several appeared only in the proteome of microfilariae, such as molecules associated with nuclear import signal receptor activity (1), lipid-binding (3) and protein binding, and bridging (2). One protein associated with 4 iron, 4 sulfur cluster binding and one related with lipid-binding appeared only in the immunoproteome of the adult stage.

**Figure 5:**
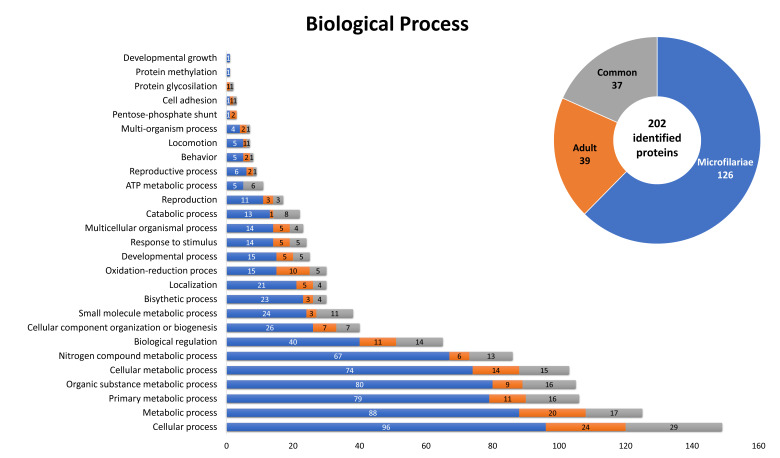
Identified *D. repens* proteins classified by biological processes, in which they may be involved, according to gene ontology (GO), The information was obtained from UniProtKB databases.

Moreover, 202 of the identified molecules were assigned by the predicted biological processes (Figure 5). A total of 126 were characteristic for microfilariae, 38 for the adult stage, and 37 were common. Most of the identified proteins were related to cellular and metabolic processes. However, members of three subcategories were presented only in the microfilariae stage: developmental growth (1), protein methylation (1), and pentose-phosphate shunt (1); and two proteins related to pentose-phosphate shunt were only present in the proteome of the adult stage.

**Figure 6:**
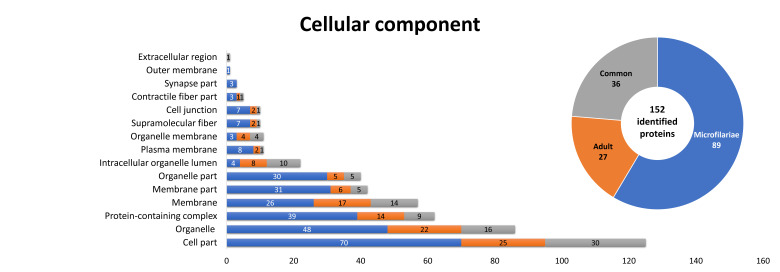
Identified *D. repens* proteins classified as a cellular component at both stages, according to gene ontology (GO). The information was obtained from UniProtKB databases.

Finally, based on the gene ontology (GO) annotation, 152 proteins were classified by cellular components, 89 specific for microfilariae, 27 for the adult stage, and 36 were common (Figure 6). Most of them were annotated as a cell part or membrane. Although both stages differ in identified proteins, there were no subcategories, members of which appeared only in one of the examined proteomes.

Three proteins (9 spots) identified in microfilariae proteome and six proteins (10 spots) from the adult stage proteome were not assigned by their molecular function or biological process or as a cellular component and were annotated as uncharacterized.

### 2.5. Molecular Cloning of Dre-33 cDNA and Drpa cDNA

Cloning of both 3′ and 5′ end cDNA of the *dre-33* gene resulted in a 705 bp product. The full-length coding sequence of the adult stage of *dre-33* was deposited in the GenBank under accession number: MH049430.1. A 655 bp partial coding sequence of microfilariae *dre-33* was deposited in the National Center Biotechnology Information (NCBI) database under accession number: MG889455.1. For the *drpa* gene, the 399 bp partial coding sequence was deposited in the NCBI database under accession number: MT063195.1.

### 2.6. Real-Time PCR Analysis of Dre-33 and Drpa Gene Expression

**Figure 7:**
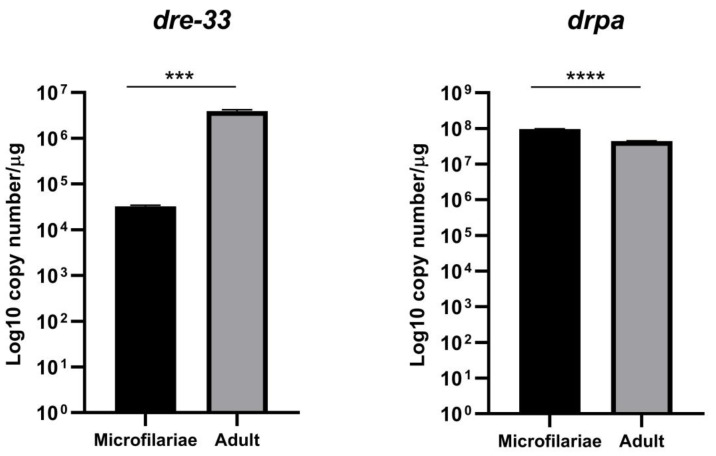
Analysis of the expression level of *dre-33* and *drpa* in microfilariae and the adult stage of *D. repens*. The result shows the number of gene copies per µg of total RNA. The bars show mean ± SEM. The statistical significant differences between examined groups are marked with asterisks: *** *p* < 0.002, **** *p* < 0.0001.

Gene expression analysis was performed to assign stage-specific genes of *D. repens*. We analyzed the expression levels of two genes coding Dre-33 (identified as immunogenic in the adult stage of *D. repens* with 1-DE and 2-DE and in microfilariae with 1-DE) and DRPA. We observed expression of the *dre-33* gene in the adult stage, which was 166 times higher than microfilariae (Figure 7). Expression of the *drpa* gene was two times higher in microfilariae stage than in the adult stage (Figure 7). Gene expression analysis indicates a higher expression of the *dre-33* gene in the adult stage than the *drpa* in the microfilariae stage.

### 2.7. Bioinformatic Evaluation of Dre-33 in Microfilariae and the Adult Stage of D. repens

The bioinformatic evaluation was performed to characterize *D. repens* protein, which was identified as potentially immunogenic and stage-specific for the adult worm. Additionally, sequences from both stages were compared to reveal some potential differences in the activity of the proteins.

**Figure 8:**
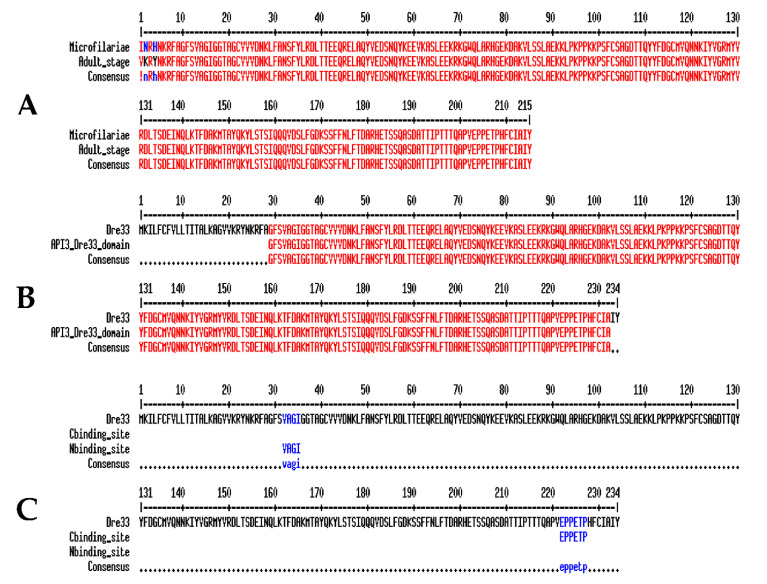
Dre-33 aa sequence characterization: (**A**) Comparison of amino acid sequences of adult and microfilariae Dre-33 (Multalin); (**B**) Dre-33 ascaris protease inhibitor 3 (API3) domain marked on the amino acid sequence (red); (**C**) N-terminal and C-terminal protease binding sites marked on the Dre-33 sequence (blue).

In both microfilariae and the adult Dre-33 sequence, encoded 234 amino acids and SignalP revealed the presence of a 19 aa signal peptide. Dre-33 contains a domain that belongs to the ascaris protease inhibitor 3 (API3) (Figure 8B) superfamily (conserved protein domain database id: cd00225). This domain plays a role in inhibiting aspartic protease, cathepsin E, gastric enzymes, pepsin, and gastricsin activity and is localized in the 29–232 position of Dre-33. Protease binding regions were identified in positions- 22–25 and 222–227 (Figure 8C). Comparison of sequences between microfilariae and the adult stage did not reveal any significant differences. The amino acid sequences differed by only three amino acids. No differences were observed in the protease inhibitor domain, protease binding sites, or predicted post-translational modifications, which might suggest that protein performs the same function in both stages of the parasite.

## 3. Discussion

The genome of *D. repens* was first sequenced de novo in 2019, predicting 10,357 genes and 11,262 proteins [32]. Comparing to *D. immitis*, the genome of *D. repens* was approximately 17% bigger, but possesses 8.9% less predicted genes. Moreover, the analysis of *D. repens* genome revealed a lower number of exons per gene and shorter average exon length than in *D. immitis*. Additional comparative transcriptomic analysis of microfilariae and L3 stage larvae indicated that 155 genes were upregulated in the L3 stage, whereas 57 were upregulated in the microfilariae stage. Gene ontology enabled assignment of 15 and 12 biological processes for the L3 and microfilariae stages, respectively [32]. De novo sequencing of the *D. repens* genome and comparative transcriptomic analysis provide an overview of selected molecular processes and potential differences between the parasite stages. This research is one of the first steps for a better understanding of parasite biology and, together with the proteomic and immunoproteomic data presented here, could be crucial to reveal potential diagnostic antigens and may help to select potential drug and vaccine targets.

With the use of 1-D and 2-D immunoblotting, supported with mass spectrometry, we examined immunoproteomes of *D. repens* adult worms and microfilariae. To our knowledge, this study represents the first simultaneous identification of immunogenic antigens of *D. repens* adult worms and microfilariae in serum collected from naturally infected dogs. Molecules that we identified in *D. repens* tissue extracts have been previously considered a potential diagnostic target, vaccine candidates, and drug targets for treating, e.g., schistosomiasis [33,34,35], cestodiasis [36,37,38,39,40,41,42], and parasitic nematodes [43,44,45,46,47,48,49,50,51,52]. Our analysis also confirmed the existence of molecules for which antigenic properties have never been considered in *Dirofilaria* genus.

We identified 316 potentially immunogenic proteins: 183 proteins were detected only in microfilariae, 75 in the adult stage, and 58 were identified as common in both stages. The previous examination of soluble extracts of *D. repens* adult worms, performed by Gonzalez-Miguel et al. [53], revealed 43 immunoreactive proteins for the adult parasite. This discrepancy may be a result of different sera used in both experiments. We performed the analysis with sera of naturally infected dogs with confirmed microfilaremia, whereas Gonzalez-Miguel used serum collected from an infected human. Humans are considered accidental hosts and, in most cases, no microfilariae are observed in the bloodstream. Absence of microfilariae in the blood and no microfilaria released by female parasites probably results in less antigen-specific antibodies common for both stages. Another factor might be the denaturing condition used to prepare parasite extracts that might have an impact on better protein recovery from both soluble and insoluble fractions.

In comparison with Gonzalez-Miguel’s results [53], two proteins were missing in our analyses (1-DE and 2-DE): transglutaminase and cyclophilin. Transglutaminases take part in the biosynthesis of the cuticle that plays a crucial role in growing and maturation of the parasite [54,55,56]. In *D. immitis*, transglutaminases were localized in the hypodermis, a fibrillar muscle cell in adult female and male worms, in the gut epithelium, and in developing embryos in females [57]. These molecules show no homology to mammalian transglutaminases and, therefore, are considered drug targets in filarial infections [54].

Cyclophilins have a wide range of functions: they are receptors for anti-parasitic agent cyclosporin A, have prolyl isomerase activity [58,59], and, in *Caenorhabditis elegans*, they are present in muscle tissues and play a role in the development of body-wall muscle cells [58].

Another difference was related to DiT33 protein, which was recognized by a serum sample from a patient with pulmonary dirofilariosis, but not with subcutaneous dirofilariosis. Interestingly, in our research, Dre-33 (as we called it, DiT33 homologue) was recognized by a dog’s serum in both stages in 1-DE analysis and the adult stage in the 2-DE analysis. We can hypothesize that the reason for the difference in results of these two experiments may be that pepsin inhibitor Dre-33 is probably related to the occurrence of microfilariae in the bloodstream. In case of *Onchocerca volvulus*, this protein was localized in hypoderm, muscle tissue, the uterus of female worms, and embryonic microfilariae [60] and, as reported by Willenbucher et al. [61], was secreted by a female worm at the amount of 10 ng per day. There are some indications that female worms may secrete this protein during the production of microfilariae. Lack of mature females and microfilariae in human hosts may result in the absence of specific Dre-33 antibodies and justifies the absence of this molecule in the 2-DE analysis by Gonzalez-Miguel and co-authors [53].

The *D. immitis* pepsin inhibitor was called a “specific and early marker of heartworm infections” by Hong et al. in 1995 [62]. It was considered that diagnosis of infection with this antigen is possible after 11 weeks of infection. We cloned *D. repens* pepsin inhibitor cDNA from both stages and performed a computational evaluation of predicted aa sequences. However, no significant differences between protein sequences from either stage were identified; the gene expression was 115× higher in adult worms when compared with microfilariae. The results lead us to an assumption that this antigen may be specific for the adult worm.

We can subdivide immunogenic proteins of *D. repens* to three major groups: structural proteins, enzymes, and chaperones. In helminths, structural proteins are most probably involved in yet undiscovered parasite-host complex interactions. These are predominantly intracellular proteins, which might be exposed to the host immune system: (1) via direct contact between the parasite body and host tissue, (2) as enzymes and other proteins released from the digestive tract, and (3) using non-classical export, as described, in different species of helminth parasites, i.e., vesicle transport [42,50,63,64].

Cytoskeleton proteins (actin, tubulin, myosin, and paramyosin) were previously found in *D. immitis* [51,53,65,66,67,68]. In the present study, cytoskeleton proteins were identified as potential antigens in both developmental stages (actin). Additionally, tubulin, myosin, and paramyosin were described as major proteins of the adult *D. repens*. Structural proteins were also recognized as potential antigens in other tissue nematode species, namely in *D. immitis* by Oleaga et al. [68] and in *Trichinella britovi* by Grzelak et al. [49]. In helminths, paramyosin was proposed to protect invading helminths from immune attack by acting as a ”decoy“ for binding proteins of the complement pathway and therefore is believed to play a role as a host immune response modulator [37]. Moreover, together with actin, it is probably involved in tegumental repair and considered one of the vaccine target molecules [69,70]. Since our immunoproteomic approach shows the presence of certain cytosolic and structural proteins as possible antigens, we suppose that the mechanism of their trafficking may have involved alternative methods of transport (e.g., excretory vesicles, exosomes, etc.). Moreover, structural proteins and/or enzymes can also be excreted from the gastrointestinal tract of the parasite or induce an immune response by direct contact of the parasite with the host tissue at both the larval and adult stages of parasite development.

Parasitic enzymes, apart from being engaged in metabolic processes, are considered to exhibit moonlighting activity, including immunomodulatory functions [42,49,51,71,72,73]. For example, GAPDH found in both *D. repens* developmental stages was identified in other helminths as a potential immunomodulator and an interesting drug target. In recent years, researchers renewed interest in targeting metabolic enzymes in the treatment of infectious diseases [74]. Our study indicates the presence of three catalytic enzymes engaged in glycolysis (enolase, fructose-bisphosphate aldolase, and glyceraldehyde-3-phosphate dehydrogenase) as essential antigens present in both developmental stages and as being spot specific. These antigens were also recognized by sera collected from *Dirofilaria*-infected humans [53]. Research on alpha-enolase from the human filarial parasite *Onchocerca volvulus* proved its role as a plasminogen receptor [75]. Additionally, studies on fructose-bisphosphate aldolase showed its engagement in minimizing the effects of the oxidative stress, as demonstrated for digenean trematodes [76]. Furthermore, together with galectins, aldolases are thought to be responsible for the specific IgE response in humans exposed to *D. immitis* [77].

Another protein of potential interest is beta-galactoside-binding lectin, belonging to galectins [78]. Galectins are involved as modulators in metabolic and inflammatory processes [79]. In human dirofilariosis, galectin and aldolase-like molecules are responsible for the specific IgE response in humans exposed to *D. immitis* [58].

In the immunoproteome of both *D. repens* stages, another dominant protein family is heat shock proteins (HSP). As previously described in helminths, HSP70 is a significant target of immune responses of the host [80,81]. HSP70 is believed to play an important role during the infection as an immunomodulator of an early humoral immune response and, therefore, may be considered a good target for immunodiagnosis [80] or a vaccine candidate [82]. Another protein belonging to the HSP family, which was identified in the immunoreactive spots of both *D. repens* developmental stages, is HSP60. In *Schistosoma japonicum*, egg-derived HSP60 is considered to induce a regulatory T-cell (Treg) [71,80]. Moreover, similar to HSP70, vaccination with HSP60 conferred protection to helminth infection in a murine model [83]. Moreover, small-HSPs (sHSPs), also recognized in the adult *D. repens*, are considered essential targets in the fight against parasitic diseases [84].

Polyprotein antigens belong to a vast family of nematode’s polyprotein antigens (NPA) [85], and homologues were found in most of the filarial worms, such as *D. immitis, Brugia malayi,* and *Loa loa* [86,87,88,89,90]. We cloned a partial coding sequence of *drpa* and assessed its expression level in both stages. We noticed a significantly higher expression of this gene in the microfilariae stage compared to the adult stage. Our analysis revealed that the *drpa* expression level was significantly higher than the other examined *dre-33* gene. The reasons for the lack of DRPA protein in our experiment are probably as follows: not enough immunogenicity of the molecule, molecule was released as an excretory-secretory product, and/or the amount of protein was not enough to detect.

## 4. Materials and Methods

### 4.1. D. repens Adult Stage and Microfilariae Tissue Lysates Preparation

Adult parasites were obtained from subcutaneous tissue of dogs during surgical procedures; microfilariae were collected from blood samples of infected dogs received from the Small Animal Hospital, Warsaw, University of Life Sciences. Microfilariae were isolated from blood using a filtration method with additional washing steps with PBS. *Dirofilaria repens* adult worms were washed with PBS to remove debris and homogenized manually in lysis buffer, containing 8 M Urea, 4% CHAPS, and 40 mM Tris, while microfilariae were suspended in lysis buffer and homogenized using TissueLyser LT (Qiagen, Hilden, NRW, Germany) and manual homogenizer. Lysates were purified with the 2-D Clean-Up Kit (GE Healthcare, Chicago, IL, USA), in accordance with the manufacturer’s protocols.

### 4.2. 1-DE, 2-DE, and Immunoblotting

Microfilariae and adult stage somatic proteins (1.2 µg of each lysate) were applied on 12% polyacrylamide gel for 1-DE separation using 1× TGS (0.025 M Tris, 0.192 M Glycine and 0.1% SDS) as the running buffer in denaturing conditions. The electrophoresis was performed at 200 V constant voltage for 45 min in Mini-Protean^®^ Tetra Cell (Bio-Rad, Hercules, CA, USA) apparatus.

Additionally, somatic proteins were resolved by 2-DE. Isoelectric focusing (IEF) was performed with immobilized pH-gradient strips (IPG-strips, pH 3–10, 7 cm, Bio-Rad, Hercules, CA, USA). The mixture of proteins (approximately 100 µg) was rehydrated in 250 μL of rehydration solution (ReadyPrep™ 2-D RehydrationBuffer, Bio-Rad) and loaded on Immobilized pH gradient (IPG) strips and incubated overnight. The following voltage steps performed IEF: 2 h at 200 V, 2 h at 400 V, and 16 h at 800 V in 20 °C. Strips were then equilibrated for 25 min by incubating in an equilibration buffer (ReadyPrep™ 2-D Starter Kit Equilibration Buffer I, Bio-Rad), followed by a 25 min incubation in the same buffer, enriched with 2.5% iodoacetamide (ReadyPrep™ 2-D Starter Kit Equilibration Buffer II).

The second dimension SDS-PAGE was run on 12% polyacrylamide gel using Mini-Protean^®^ Tetra Cell (Bio-Rad) apparatus at 200 V constant voltage for 45 min.

Separated proteins were stained with a Silver Staining Kit, according to the manufacturer’s protocols (Krzysztof Kucharczyk Techniki Elektroforetyczne, Warsaw, Poland) or used for Western blot analysis. For immunoproteomic purposes, proteins were electrotransferred from 2-D gels to nitrocellulose membranes, followed by blocking in protein Pierce™ Protein-Free T20 Blocking Buffer (Thermo Fisher Scientific, Waltham, MA, USA). Membranes were washed with PBS and incubated with serum from an infected dog at a dilution of 1:5000 for 1 h. Next, membranes were washed and incubated with the anti-dog-IgG secondary antibody, conjugated to horseradish peroxidase (HRP) (Sigma-Aldrich, St. Louis, MO, USA) at a dilution of 1:10,000 for 1 h. The 2-D blots were developed with SuperSignal West Pico Chemiluminescent Substrate (Thermo Fisher Scientific), according to the manufacturer’s protocols. The experiment was performed with two replicates.

### 4.3. LC-MS/MS Identification and Bioinformatics

For further analysis, only bands and spots present in both SDS-PAGE and immunoblot were chosen. Selected fragments were manually excised from the silver-stained gels and subjected to LC-MS/MS in the Laboratory of Mass Spectrometry, Institute of Biochemistry and Biophysics, Polish Academy of Sciences (Warsaw, Poland). Samples were subjected to the standard “in-gel digestion” procedure, in which they were first dehydrated with acetonitrile (ACN) and then reduced, alkylated, and digested with trypsin, as previously described by Kordan et al. [91]. Briefly, the gel pieces were first treated with 10 mM DTT in 100 mM NH_4_HCO_3_ for 30 min at 57 °C and then with 0.5 M iodoacetamide in 100 mM NH_4_HCO_3_ (45 min in the dark at room temperature). Proteins were digested overnight with 10 ng/μL trypsin in 25 mM NH_4_HCO_3_ at pH 8.5 (Promega, Madison, WI, USA) at 37 °C. The resulting tryptic peptides were extracted in a solution containing 0.1% formic acid and 2% ACN. Samples were concentrated and desalted on an RP-C18 pre-column (Waters Corporation, Milford, MA, USA), and further peptide separation was achieved on a nano-ultra performance liquid chromatography (UPLC) RP-C18 column (Waters, BEH130 C18 column, 75 μm i.d., 250 mm long) of a nano ACQUITY UPLC system, using a 45-min linear acetonitrile gradient. The column outlet was directly coupled to the electrospray ionization (ESI) ion source of the Orbitrap Velos type mass spectrometer (Thermo Fisher Scientific), working in the regime of data-dependent MS to MS/MS switch with high-energy collision dissociation (HCD) type peptide fragmentation. An electrospray voltage of 1.5 kV was used. Raw data files were pre-processed with Mascot Distiller software (version 2.5, Matrix Science Inc, Boston, MA, USA).

The obtained peptide masses and fragmentation spectra were matched to the National Center Biotechnology Information (NCBI) non-redundant database, with a Filarioidea filter using the Mascot search engine (Mascot Server v. 2.4.1, Matrix Science). The following search parameters were applied: enzyme specificity was set to trypsin, peptide mass tolerance to ±30 ppm, and fragment mass tolerance to ±0.1 Da. The protein mass was left as unrestricted, and mass values as monoisotopic with one missed cleavage was allowed. Alkylation of cysteine by carbamidomethylation as fixed and oxidation of methionine was set as a variable modification.

Multidimensional Protein Identification Technology–type (MudPIT-type) and/or the highest number of peptide sequences were selected. The expected value threshold of 0.05 was used for analysis, which means that all peptide identifications had a <1 in 20 chance of being a random match. Spectra derived from silver-stained gel pieces usually do not contain enough MS/MS fragmentations to calculate a meaningful FDR; therefore, a Mascot score threshold of 30 or above (*p* < 0.05) was used.

Mascot data analysis resulted in a list of identified proteins for every analyzed sample (gel fragment). Each identified protein was described by multiple parameters: score, matches, sequences, emPAI, and protein sequence coverage. Proteins selected for analysis were with matches and sequences above or equal to 4%, and protein sequence coverage was above or equal to 5%.

The identified proteins were classified according to their predicted molecular function, biological process, and cellular component using the UniProtKB database (http://www.uniprot.org/ (accessed on 20 December 2020), The UniProt Consortium, 2018). In a part of our analysis, we selected the molecules identified by both the 1-DE and 2-DE methods in each of the parasite stages (Table 1).

### 4.4. Cloning of cDNAs Encoding Dre-33 and Drpa Genes and Bioinformatic Evaluation

Total RNA was isolated from adult worm and microfilariae using Total RNA Mini Kit (A&A Biotechnology), treated with DNase I (Thermo Fisher Scientific), and used as a template in reverse transcription reaction with RevertAid First Strand cDNA Synthesis Kit (Thermo Fisher Scientific) and PTX primer (5′ GAA CTA GTC TCG AGT TTTTTTTTTTTTTTTTTT 3′). All procedures were performed according to the manufacturer’s protocols. cDNA encoding *dre-33* was cloned using the RACE–PCR method, as previously described [92,93,94,95]. The 3′ end of cDNA was amplified using PTX primer and gene-specific primer For3′_dre33 (5′ GAT GGY TGY ATG GTT CAG AAT 3′, where Y is a degenerate nucleotide). The reaction was performed under the following conditions: 3 min at 95 °C, 35 cycles of: 95 °C for 30 s, 50 °C for 45 s, and 72 °C for 40 s, with a 10 min final extension step at 72 °C. The reverse transcription product was enriched in polyC tail by terminal deoxynucleotidyl transferase (TdT) and used as a template in 5′ RACE–PCR reaction. The 5′ end of *dre-33* cDNA was amplified with the poliGEcoRI primer (5′ CGA GGA ATT CGG GGG G 3′) and Rev5′_dre33 gene-specific primer (5′ TTG TTG TTTC ACG TTG ATC ATA 3′) under the following conditions: 3 min at 95 °C, 35 cycles of: 95 °C for 30 s, 51 °C for 45 s, and 72 °C for 40 s, with a 10 min final extension step at 72 °C. Both ends were ligated into pGEM–T Easy vector and sequenced by Sanger’s method to confirm the specificity of cloned products (Genomed S.A., Poland). To amplify the entire coding sequence of the *Dre33* gene, new primers were designed: For_dre33_cds (5′ ATG AAA ATT CTT TTT TGC TTT GTG C 3′) and Rev_dre33_cds (5′ TCA ATA AAT TGC AAT ACA GAA ATG T 3′) and used in the reaction under conditions, as follows: 5 min at 95 °C, 35 cycles of: 95 °C for 30 s, 50 °C for 45 s, and 72 °C for 45 s, with a 10 min final extension step at 72 °C. Finally, the full length 705 bp PCR product was ligated into the pGEM–T Easy Vector and confirmed by sequencing.

Based on the confirmed adult sequence, the partial coding sequence of microfilariae *dre-33* cDNA was cloned. For_dre33_micro (5′ TAG CGT CAT AAA TCG ACA C 3′) and Rev_dre33_micro (5′ TCA ATA AAT TGC AAT ACA GAA ATG T 3′), primers were used to amplify cDNA encoding only in the mature part of the antigen (without signal peptide). Conditions of the reaction were the same as described previously.

The partial coding sequence of *drpa* cDNA was amplified with For_drpa (5′ TGC TTG ATG GCT CTC AAT GAA ATT 3′) and Rev_drpa (5′ ACC TTG TTG AAG TTC TTC AGC TG3′) primers under the following conditions: 5 min at 95 °C, 35 cycles of: 95 °C for 30 s, 5 °C for 45 s, and 72 °C for 30 s, with a 10 min final extension step at 72 °C. The following procedures were conducted, as described previously. All gene-specific primers were designed based on the homologous genes in the closely related filarial worm.

Alignment of adult and microfilariae aminoacid sequences of Dre-33 was created in Multalin [96], and SignalP predicted the potential signal peptides [97].

### 4.5. Real-Time PCR Analysis of Dre-33 and Drpa Gene Expression

Analysis of gene expression was performed on mRNA extracted from adult *D. repens* worms and microfilariae. Total RNA isolation, DNase treatment, and reverse transcription were performed as previously described. Real-time analysis was conducted in 96-well PCR Plates (Applied Biosystems, Foster City, CA, USA) in a QuantStudio6 Real-Time PCR system (Applied Biosystems). In total, 10 ng of reverse transcription mixture was used as a PCR template to determine the gene expression level. PowerUp SYBR Green Master Mix (Applied Biosystems) was used with For_dre33RT (5′ CTG AAG AGC AAC GAG AAC TTG CAC AA 3′) and Rev_dre33RT (5′ TGT CGT GCT AAT TGC CAT CCT TTA CG 3′) primers for *dre-33* analysis and For_drpa_RT (5′ CGG AGG AAT CAG AAT GAA AGT CGA AG 3′) and Rev_drpa_RT (5′ CGT GCA TTC ATT GCC GCA TAG ATT TTA C 3′) for *drpa* analysis. Reactions were performed by a two-step procedure according to the manufacturer’s protocol, including the dissociation curve step. A standard curve (1 × 10^1^ to 1 × 10^6^ copies per reaction) was used to determine the number of *Dre33* and *DRPA* gene copies. The data collected during the anneal/extend step was analyzed for statistically significant differences between groups using the Student’s *t*-test.

## 5. Conclusions

The microfilariae and the adult *D. repens* immunoproteome characterization with the use of 1-D and 2-D immunoblotting is hereby reported. Identification of stage-characteristic proteins linking to biological processes and molecular functions is crucial for better understanding of molecular mechanisms occurring between parasite and host. These include strategies of invasion and escaping from the host immunological system. All this may illustrate evolutionary adaptations to the parasitic way of life and general biology of *D. repens*. The identified immunodominant molecules belong to chaperones, structural and enzymatic proteins, suggesting their roles in mediating the interactions between the parasite and the host. Identification of structural and enzymatic proteins may indicate their moonlighting activity in the complex parasite-host interplay. Recognition of stage-specific and common proteins may help to select the most promising candidates for serological diagnostics and vaccines. Moreover, these results may be useful in the new drug targets discovery. Therefore, we believe that our data will contribute to a better understanding of the biology of this zoonotic pathogen and development of strategies to prevent its spread.

## Figures and Tables

**Table 1:** With both techniques (1-DE and 2-DE), immunogenic proteins identified electrophoresis in adults and microfilariae *D. repens.*

NCBI ID	UniProt ID	Protein Name	Adult	Microfilariae
AHJ11156.1	W6E957	14-3-3 zeta	+	+/−
EJW88499.1	J9FM82	26S protease regulatory subunits	+	+/−
AAD13153.1	P90689	Actin	+	+
EJW88625.1	J9BM73	Actr1a-prov protein	+	−
OZC12423.1	A0A183HZF6	ATP synthase subunit alpha	−	+
CRZ24515.1	A0A0H5S7C6	Bm13662, isoform a	−	+
P37801.2	A0A044V456	Calponin homolog OV9M	+	−
XP_003136825.1	A0A0B2VPC7	Calponin -like protein OV9M	+	+
EJD76193.1	A0A1S0UKZ6	Calponin OV9M	+	+/−
P11012.2	P11012	Calreticulin (41 kDa larval antigen) (Protein ral-1)	+	+/−
EJD73707.1	A0A1S0UFQ7	CBR-PQN-22 protein (Fragment)	+	−
EJD73722.1	A0A1S0UDI6	CBR-UNC-54 protein	+	−
OZC11261.1	A0A238C1A7	Chaperone protein DnaK	−	+
AAD27589.1	Q9XYR7	Chaperonine protein HSP60	+	+
AAA74283.1	Q25616	Circulating antigen	+	+/−
CDP96739.1	Q17049	Cytoplasmic intermediate filament protein	+	+/−
AAA85099.1	Q25598	Disulfide-isomerase (EC 5.3.4.1)	+/−	+
OZC08156.1	A0A183H0T4	EF hand	+/−	+
AHI18146.1	W5XWA0	Enolase	+	+
AFL46381.1	I3WTW4	Fructose-bisphosphate aldolase (EC 4.1.2.13)	+	+
EFO26688.2	A0A1S0U7X9	Galectin	+	+
AFL46382.1	I3WTW5	Glyceraldehyde-3-phosphate dehydrogenase (EC 1.2.1.12)	+	+
CAA70570.1	P91886	Heat shock protein 60	+	+
AAD13154.1	O96541	Heat shock protein 70	+	+
CAA06694.1	O61998	Heat shock protein 90	+	+/−
AAF37702.1	Q9NGY2	Intermediate filament protein	+	+
XP_003138426.1	A0A1I7VW91	Intermediate filament protein ifa-1	+	+/−
OZC09651.1	A0A238BWF8	Intermediate filament tail domain protein	+	+
OZC04931.1	A0A238BHT0	KH domain protein	−	+
XP_003140990.1	A0A1S0U036	LFI-1 protein	+	−
EJD74046.1	A0A1I7VEY5	Major antigen	+	−
AAA29420.1	Q04010	Major body wall myosin	+	−
Q17107.1	Q17107	Muscle cell intermediate filament protein AV71 (Fragment)	+	−
CRZ23584.1	P31732	Muscle cell intermediate filament protein OV71	+	+/−
XP_001899601.1	A0A0K0JD94	Myosin heavy chain	+	−
EJD76606.1	A0A1S0UMD2	Myosin tail family protein	+	−
EJW85566.1	J9ESQ7	Paralyzed arrest at two-fold protein 6	+	+/−
P13392.2	P13392	Paramyosin (Fragment)	+	−
Q27384.1	Q27384	Pepsin inhibitor DiT33	+	+/−
XP_003141782.1	A0A1I7V808	Phosphoglycerate kinase (EC 2.7.2.3)	+	+/−
XP_001897696.1	A0A0K0JQX1	RNA recognition motif containing protein	+/−	+
EJD76743.1	A0A1I7VNT2	Spectrin protein 1	+	−
EJD74006.1	A0A1S0UGM3	T-complex protein 1 subunits	+	+
EFO19766.2	A0A1I7VRC2	TPR domain-containing protein	+	+
EJD76054.1	A0A1S0UML3	Transitional endoplasmic reticulum ATPase 1	+	−
EJD75137.1	A0A1S0UJV8	Tropomyosin	+	+
OZC10791.1	A0A238BZW2	Troponin	+	+/−
XP_003145669.1	A0A1I7VMU9	Troponin T	+	+
XP_020302030.1	A0A0K0JWL2	Tubulin beta	+/−	+
EFO16380.2	A0A0B2VMC0	Type I inositol 1,4,5-trisphosphate 5-phosphatase	+	+/−
XP_003139291.1	A0A1I7VJS5	Ubiquitin-conjugating enzyme E2 N	−	+
OZC06791.1	A0A238BMY9	WD domain, G-beta repeat protein	−	+

“+”—identified with 1-DE and 2-DE; “+/−”—identified only with one method; “−”—no identification.

## Data Availability

The data presented in this study are available in [insert article or supplementary material here].

## Supplementary Materials

The following are available online at https://www.mdpi.com/2076-0817/10/2/174/s1, Supplementary File S1: Results of the LC-MS/MS analysis of immunoreactive protein bands of the microfilaria *D. repens* proteome, recognized by sera from *D. repens* infected dogs. Each sheet represents a different immunoreactive band. Supplementary File S2: Results of the LC-MS/MS analysis of immunoreactive protein bands of the adult *D. repens* proteome, recognized by sera from *D. repens* infected dogs. Each sheet represents a different immunoreactive band. Supplementary File S3: Results of the LC-MS/MS analysis of immunoreactive protein spots of the microfilaria *D. repens* proteome, recognized by sera from *D. repens* infected dogs. Each sheet represents a different immunoreactive spot. Supplementary File S4: Results of the LC-MS/MS analysis of immunoreactive protein spots of the adult *D. repens* proteome, recognized by sera from *D. repens* infected dogs. Each sheet represents a different immunoreactive spot.
